# Privacy-Preserving Indoor Trajectory Matching with IoT Devices

**DOI:** 10.3390/s23084029

**Published:** 2023-04-16

**Authors:** Bingxian Lu, Di Wu, Zhenquan Qin, Lei Wang

**Affiliations:** School of Software Technology, Dalian University of Technology, Dalian 116024, China; bingxian.lu@dlut.edu.cn (B.L.);

**Keywords:** IoT security, Wi-Fi, privacy protection, homomorphic encryption

## Abstract

With the rapid development of the Internet of Things (IoT) technology, Wi-Fi signals have been widely used for trajectory signal acquisition. Indoor trajectory matching aims to achieve the monitoring of the encounters between people and trajectory analysis in indoor environments. Due to constraints ofn the computation abilities IoT devices, the computation of indoor trajectory matching requires the assistance of a cloud platform, which brings up privacy concerns. Therefore, this paper proposes a trajectory-matching calculation method that supports ciphertext operations. Hash algorithms and homomorphic encryption are selected to ensure the security of different private data, and the actual trajectory similarity is determined based on correlation coefficients. However, due to obstacles and other interferences in indoor environments, the original data collected may be missing in certain stages. Therefore, this paper also complements the missing values on ciphertexts through mean, linear regression, and KNN algorithms. These algorithms can predict the missing parts of the ciphertext dataset, and the accuracy of the complemented dataset can reach over 97%. This paper provides original and complemented datasets for matching calculations, and demonstrates their high feasibility and effectiveness in practical applications from the perspective of calculation time and accuracy loss.

## 1. Introduction

With the rapid development of IoT technology, more and more IoT devices are being used in various fields. IoT technology provides wireless communication, data transmission, and data processing capabilities for IoT devices, allowing these devices to collect and analyze large amount of data. These IoT devices have access to relevant data in areas such as social networking [1,2,3], intelligent transportation, and medical monitoring. These data can be used to analyze users’ location information [4,5,6], health condition [7], etc., which can provide more accurate and timely services.

IoT devices can collect trajectory data by scanning the surrounding Wi-Fi signals [8,9]. Trajectory matching refers to uploading user-generated trajectory data to a cloud server for processing and analysis to match the user’s travel trajectory and performing encounter detection and trajectory analysis for them. Wi-Fi networks [10,11,12] are widely used for their easy deployment, low cost, and fast information transmission rate and because a variety of terminals can be accessed. Therefore, indoor trajectory matching based on Wi-Fi signals is a research direction worthy of attention.

Due to the immense quantity of indoor trajectory matching calculations, it is impossible to compute trajectory using IoT devices. The outsourced computing provided by cloud computing [13,14,15,16] service providers can improve the speed and efficiency of the calculation [17], but the most important concern of users is data security when using these services. Trajectory data contain the users’ location information and movement trajectory; the leaking or malicious use [18] of these may lead to the user’s personal privacy being violated, such as the user being followed or their personal information being leaked. Even if the user encrypts the original data, a decryption operation is required when performing the calculation. Therefore, the security [19,20,21] of the data calculation process cannot be guaranteed.

Initial IoT devices lack privacy protection features. As a result, data such as users’ trajectory data and location information are widely shared, which posed a significant privacy risk. As users become more concerned about data privacy, more and more IoT devices are beginning to support privacy protection features. For example, users’ trajectory information can be protected in smartphones by turning off or restricting location sharing. Later, to protect users’ trajectory information, some encryption or anonymization techniques have started to be used in the devices. When using encryption algorithms, only authorized users can access the data, which protects the user’s trajectory information. Federal learning technology spreads the user’s trajectory data to different devices for processing, which protects the privacy and security of the user’s data.

In recent years, several studies have argued that the most popular technique to achieve the desired location privacy protection is to reduce the resolution of location information, i.e., allowing the use of hidden spatial regions to obfuscate the exact location of the user, such as in k-anonymity-based methods [22]. However, the processing of the dataset in k-anonymization algorithms causes distortion and blurring of the data, which compromises the accuracy and continuity of the trajectory. Therefore, in terms of privacy security of trajectory matching, accurate matching of trajectories cannot be achieved using k-anonymity methods. In addition, Chunyong Yin proposed a location privacy protection method satisfying differential privacy constraints in [23], where a multi-level location tree model was established to ensure the privacy of the data. However, in the framework of differential privacy, the location information needs to be randomized, such as by adding noise or perturbing the location information. This will cause the correlation between trajectories to be broken so that the accuracy of trajectory matching will be seriously affected.

So far, research on applications related to homomorphic encryption [24] is being actively conducted both at home and abroad. A homomorphic encryption algorithm is a special kind of encryption algorithms since it allows for addition and multiplication operations to be performed in the ciphertext state. Trajectory matching requires complex calculation and the comparison of multiple trajectories, and traditional encryption methods require continuous encryption and decryption operations during the calculation process. In contrast, homomorphic encryption can perform calculation in the encrypted state, avoiding the overhead of decryption and re-encryption, improving the calculation efficiency while at the same time ensuring the integrity and correctness of the calculation results, and avoiding data loss and calculation errors caused by encryption and decryption operations.

Inspired by the above discussions, this paper provides a solution for indoor trajectory matching under privacy protection, i.e., achieving trajectory similarity measurement using homomorphic encryption [25]. The Pearson correlation coefficient can represent the correlation between data well, and it is thus reasonable to use it to represent the similarity between trajectory data.

IoT devices can detect the presence of wireless devices around them at any time, recording each signal simultaneously, which helps collect the user’s trajectory data at all times. However, some of the collected dataset may be missing due to the influence of obstacles indoors, for example. In this study, three ciphertext data completion methods are proposed to solve this problem. Finally, the effectiveness of the study is verified by experimental validation while guaranteeing computational accuracy. The main contributions of this study are as follows:

- A Pearson similarity coefficient calculation method based on homomorphic encryption is proposed, and the accuracy loss of the ciphertext is compared.
- Several missing value completion schemes based on homomorphic encryption are proposed to improve the accuracy of the similarity measurement.
- The effectiveness of the proposed method is verified by field data collection and numerical analysis and experiments.

The remainder of this paper is organized as follows. In Section 2, the literature related to indoor positioning and location privacy is described. An overview of the scheme is given in Section 3. Section 3.1 describes the process of data collection. Section 3.2 describes the encryption processing of the trajectory data. The Pearson similarity calculation method based on homomorphic encryption is described in detail in Section 3.3. This section also describes the properties and performance of several missing value complementation schemes based on homomorphic encryption. Section 4 gives the experimental process of the whole study and an analysis of the final experimental results. The relevant discussion is presented in Section 5. Section 6 is the conclusion.

## 2. Related Work

The overall work of this study is mainly carried out by matching users’ trajectory data while ensuring privacy and security. Therefore, the study involves knowledge in the fields of indoor positioning, location privacy, etc.

### 2.1. Indoor Location Positioning

In terms of location positioning, the existing research is relatively mature. While outdoor satellite positioning systems, such as GPS, Galileo, BeiDou, etc., have high accuracy positioning capabilities, these signals are very poorly received indoors. Therefore, many people have devoted themselves to the research of indoor positioning. At present, the positioning technologies often used in the field of indoor positioning are Wi-Fi, Bluetooth, ZigBee, ultra-wideband, radio frequency identification (RFID) devices, vision sensors, etc. The advantages and disadvantages of these indoor positioning signal source are shown in Table 1.

Using these techniques, researchers have conducted a number of related studies, for example, 3D real-time indoor localization with centimeter-level accuracy through broadband nonlinear backscattering in passive devices [26], which involves RFID localization technology; using unmodified fluorescent lamps as location landmarks and commercial smartphones as light sensors for localization [27], and so forth [28]. Each of these studies provides the theoretical and experimental basis for the development of indoor localization.

In this study, wireless signal strength is used for localization. Because wireless signals have become ubiquitous in recent years, Wi-Fi coverage has been achieved basically everywhere, including university campuses, company interiors, stations and airports, and large shopping malls. In addition, people carry mobile devices that can also turn on hotspot services to provide wireless network access to other mobile devices. At the same time, the signal interference in a location is basically the same and does not affect the results of trajectory matching. Therefore, choosing Wi-Fi signals for indoor trajectory matching is the quickest and most convenient positioning method.

### 2.2. Location Privacy

Indoor positioning can obtain information about a person’s location and facilitate subsequent location data similarity exclusion work. However, this may expose users’ location information to the public, creating the risk of privacy leakage. Therefore, securing private data should be guaranteed for users who have submitted their location data. The advantages and disadvantages of location privacy-related algorithms are shown in Table 2.

Several studies have argued that the most popular technique to achieve the desired location privacy protection is to reduce the resolution of location information, i.e., allowing the use of hidden spatial regions to obfuscate the exact location of the user, such as k-anonymity-based methods [22]. Another common approach in location privacy is called the differential privacy approach model. The differential privacy model is a powerful privacy concept that does not depend on the attacker’s background knowledge and computational power and has become a research hotspot in recent years. Differential privacy is a common privacy-preserving framework, supported by solid mathematical theory, that provides privacy protection for data when the attacker has the maximum amount of background knowledge. Differential privacy strategies [23] are insensitive to background knowledge and the DP-k model offers better protection. Laplacian and indexing mechanisms are the main implementation mechanisms of differential privacy, and the degree of privacy protection can be shown by assigning a privacy budget which is relatively reliable and strict. In [29], the function mechanism is introduced again, in which the objective function of the ξ-differential privacy interference optimization problem is used to protect privacy. The DiffP-C4.5 and DiffGen algorithms are used in [30], which ultimately allows differential privacy to be combined with decision trees and other data structures to ensure both the privacy and availability of the data. In [31], a new definition of differential privacy based on the “δ-location set” is proposed to account for temporal correlations in location data.

The special characteristics of homomorphic encryption make it more difficult to implement in practice, so it is not particularly widely used at present [32]. However, the properties of homomorphic encryption allow certain scientific research to be conducted more smoothly and efficiently [33]. In [34], an encryption-based secure SC framework is proposed, which ensures that the worker’s location information is never released to any party, but the system can still assign tasks to the workers close to each task location. Among other things, it is the use of homomorphic encryption that solves the challenge of assigning tasks based on encrypted data.

## 3. Materials and Methods

**Figure 1:**
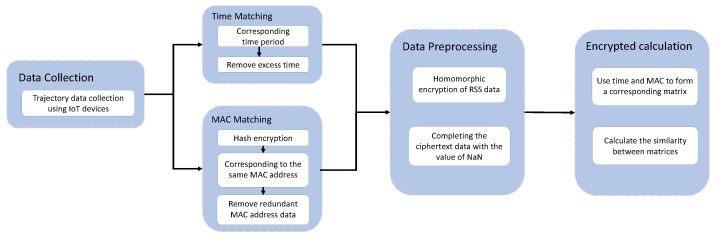
Overall flow chart of the study.

This paper is a study of the anonymous matching of indoor trajectories based on Wi-Fi signals. The study includes three parts: data collection, data processing, and trajectory matching calculation. The overall flow chart is shown in Figure 1. Data collection is performed using IoT devices. Then, the collected data are processed. Firstly, the time and MAC address are matched. Then, the RSS signal is homomorphically encrypted. When the problem of missing values is faced, the missing values need to be completed first. Finally, the Pearson correlation coefficient between the processed data is calculated to obtain the final matching result.

**Figure 2:**
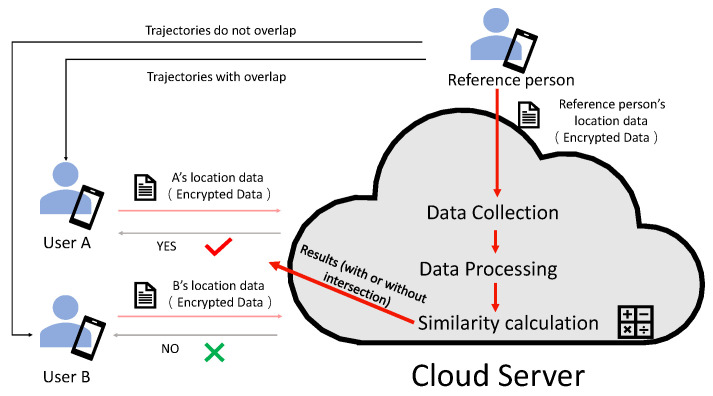
Data Interaction between IoT Devices and Cloud Services.

The solution of indoor trajectory matching based on privacy protection contains two parts: the client and the cloud computing server. The client will the collect RSS signal as the trajectory data, and when the client uploads the trajectory data to the cloud computing server, the cloud computing server will perform the calculation of trajectory matching instead of the client. For data privacy and security, the original data will be encrypted before being uploaded to the cloud computing server. In this way, when outsourcing the trajectory matching calculation to the cloud environment, the cloud environment does not receive the original data and ensures the privacy security of the client. The data interaction between the client and the cloud server is shown in Figure 2.

### 3.1. Data Information Collection

In this section, the main work performed is the collection of experimental data. The experimental data mainly include the time of data collection, the MAC addresses of the devices from which the wireless signals are emitted, and the RSS received by the IoT devices. The data collection is performed by the IoT devices (e.g., cell phones, etc.), and then data analysis is performed based on the collected results.

#### 3.1.1. Collecting Data Information

Nowadays, Wi-Fi is everywhere. People carry IoT devices, such as cell phones, that can receive RSS signals from routers or other hotspots in different directions around them. When people are in different locations, the strength of the received wireless signal is not the same when subjected to distance, direction, obstructions, and other factors [37,38]. However, the main purpose of this study is to find people who pass through the same area and are close to each other [39] within the same time period. Therefore, signal errors caused by obstacles or moving people or objects can simultaneously affect the signal results collected by people in the same location. So, the final matching result is basically not affected by these errors.

This section provides dataset support for the whole study and designs a Wi-Fi signal acquisition system based on mobile devices. The system is mainly for Android mobile smart terminals and is based on the Android Studio integrated development environment. This APP is designed to realize functions such as trajectory data acquisition and uploading.

The APP’s operation of obtaining the list of Wi-Fi networks scanned on the mobile device is established through the WifiManager service. Here, the Wi-Fi list can be managed using getWifiList (), and the RSS dataset can also be divided using getLevel (int level), where ≥−50 dBm is excellent, ≥−70 dBm is good, ≥−80 dBm is average, and ≥−100 dBm is the worst.

In the process of running the APP, the APP will continuously retrieve the Wi-Fi list scanned by the mobile device. After that, data on time, SSID, MAC address, RSS, etc., will be packaged into a dataset, and HTTP will be used to establish contact with the server through three handshakes. The Tomcat server 8080 interface will be used to upload the dataset to the server side regularly, at which point the acquisition of user trajectory data is completed.

#### 3.1.2. Handling Data Information

(1) Time Handling

Time is an important condition for performing subsequent similarity matching, and its interval should be carefully chosen. If the time interval is long, no information about a person in a certain place is captured in a certain time period, and there will be missing information. Ideally, the shorter the time interval, the better the effect of obtaining complete trajectory data. However, in practice, obtaining complete data is also influenced by the equipment side of data collection and the surrounding factors. Therefore, the interval between data collection is set to 1 s for balance.

(2) MAC Address Handling

Devices such as routers searched in different locations are different, and they all have their own unique MAC addresses. To match the MAC addresses in the data information of two or even more people, it is very time-consuming to directly compare the data one by one for the query. MAC addresses are by nature a combination of numbers, letters, etc., and are all converted to numbers after encryption. Therefore, it is possible to sort the pure numeric sequences and compare the sorted MAC addresses. This operation seems to be unchanged in terms of time complexity, but it actually reduces the number of comparisons by almost half. The method minimizes computational difficulty and finds tight links more efficiently.

### 3.2. Data Information Encryption Processing

In this section, encryption processing of data information, including the encryption of MAC addresses with a hash algorithm and the encryption of RSS with a homomorphic encryption algorithm, is presented [40,41].

#### 3.2.1. MAC Address Hiding

The collected data contain the MAC addresses and the corresponding detected RSS signal. MAC addresses cannot be directly involved in the operation of integer or floating point types of data. In this study, the purpose is to match the data information of multiple people with the same MAC address in the same time period [42]. After comparing various encryption methods, the hash algorithm was determined to be the most suitable.

The same data are hashed to obtain the same hash value, which helps with future matching. In terms of data security, hashing algorithms are irreversible. In this study, MAC address decryption will not be carried out, so hash encryption is the most appropriate choice.

For the hashing algorithm of the MAC address, SHA-256 [43] was chosen in this study. This algorithm is a secure hashing algorithm. It is characterized by fixed-length output and irreversibility. Breaking down the SHA-256 algorithm, it mainly includes modules such as constant initialization, information preprocessing, and the use of logical operations. In constant initialization, the SHA-256 algorithm [44] uses 8 hash primitives and 64 constants. These constants are obtained by taking the first 32 decimal parts of the prime numbers in the natural numbers after square or cube operations. The operations involved in SHA-256 hash functions are all logical bit operations. The main logical functions used are shown in Equations (1) and (2):

(1) $$\sigma_{0\left(x\right)}=S^{7}\left(x\right)⨁S^{18}\left(x\right)⨁R^{3}\left(x\right)$$

(2) $$\sigma_{1\left(x\right)}=S^{17}\left(x\right)⨁S^{19}\left(x\right)⨁R^{10}\left(x\right)$$

Among them, ⨁ means bitwise “Exclusive OR”, $S_{n}$ means a cyclic shift to the right by n bits, $R_{n}$ means a right shift by n bits. Equations (1) and (2) are used to move the bits and heterogeneous calculations to complete the subsequent cyclic encryption.

The MAC address is encrypted by the SHA-256 algorithm using the following computational steps: First, the message is decomposed into blocks of 512-bit size, and bit padding is performed if a block is insufficient. Next, iterative processing is performed using the mapping function Map, represented by $Map\left(H_{i-1}\right)=H_{i}$. In $Map\left(H_{i-1}\right)=H_{i}$, the first 16 words $\left[W_{1},W_{2}...W_{16}\right]$ are obtained directly from the decomposition of the i-th block of the message. The rest of the words $W_{t}$ are derived according to the following iterative formula:

(3) $$W_{t}=\sigma_{1}\left(W_{t-2}\right)+W_{t-7}+\sigma_{0}\left(W_{t-15}\right)+W_{t-16}$$

The mapping $Map\left(H_{i-1}\right)=H_{i}$ contains 64 encryption cycles. The encryption loop involves specific operations such as summing, taking the remainder, obtaining the secret key, cutting the block, looping, etc. The eight words generated by the last loop together are the hash string $H_{i}$ corresponding to the *i*-th block.

The above SHA-256 algorithm is used for the completion of the encryption of the MAC address in the  trajectory data collected by the cloud server.

#### 3.2.2. Wireless Signal Strength Encryption

Data privacy has become a key concern for contemporary society, especially when it comes to sensitive data such as healthcare data, and the cloud is not yet fully trustworthy. In this study, the data collected on the IoT devices containing trajectory data information included RSS in addition to MAC addresses. Therefore, privacy protection mechanisms must be set up for RSS to ensure the security of these data in the cloud space. In homomorphic encryption [45,46], operations such as addition and multiplication can be performed even when data are in the encrypted state. Therefore, it has recently been widely used to solve some classical privacy-preserving problems. For example, the private information retrieval and private set intersection problems are solved well based on HE in IEEES&P 2018 and ACM CCS 2017 [47]. The next stage in this study is to use RSS in the ciphertext state for the computation. Since homomorphic encryption allows the manipulation of encrypted data, this encryption scheme is the most appropriate choice for RSS processing.

The most basic security feature of homomorphic encryption is semantic security. Specifically, the ciphertext does not reveal any information in plaintext. The formula is expressed as follows:

(4) $$\forall m_{0},m_{1},Encrypt\left(PK,m_{0}\right)\approx Encrypt\left(PK,\;m_{1}\right)$$

In the above equation, PK represents the public key and *m* represents the plaintext. In addition, the sign of approximate equality implies that the polynomials are indistinguishable. Even if $m_{0},\;$$m_{1}$, and PK are known, an efficient algorithm to distinguish the two results does not exist. Even if the same plaintext is encrypted, the results obtained are different. Because there is a random number in the encryption algorithm, the privacy and security of the plaintext is guaranteed.

In this study, due to the large amount of trajectory data, cloud space is needed for data storage and operation. Here, the CKKS scheme in homomorphic encryption is selected. The CKKS scheme consists of the following seven parts: initialization, key generation, encryption, decryption, addition, multiplication, and rescaling. The process of encryption and decryption in a cloud scenario has the following steps:

(1) The parameter λ is a safety-level parameter, and *L* represents the upper depth limit. Choose a power *N* of 2, a cardinal number p<0, and a special modulus *P* (for rescaling). Define $Q=q_{0}\cdot p^{L}$ such that *N* and P·Q, satisfying the security level of λ. Choose a private key-related distribution of $\chi_{s}$, an error distribution of $\chi_{e}$, and a random distribution of $\chi_{r}$ for encryption. Use the lowercase letter *q* to denote the modulus of any layer; *q* is the approximate number of *Q*.
(2) The RSS is encrypted and sent to the cloud server. In the cloud server, the key generation function, i.e., the KeyGen function, should be run by the smartphone. This function is used to generate the key for the data to be encrypted.

Instantiate $s\leftarrow \chi_{s}$ and $a\leftarrow R_{Q}$ and set the private key $sk\leftarrow \left(1,\;s\right)$ to calculate the public key, as follows:

(5) $$pk\leftarrow \left(b,\;a\right)\in R_{Q}^{2}$$

(6) b=−a·s+emodQ

Instantiate $a^{{}^{\prime }}\leftarrow R_{PQ}$ and $e\leftarrow \chi_{e}$, and the following can be used to set the auxiliary calculation key, which is the evaluation key:

(7) $$evk\leftarrow \left(b^{{}^{\prime }},\;a^{{}^{\prime }}\right)\in R_{PQ}^{2}$$

(8) $$b^{{}^{\prime }}=-a^{{}^{\prime }}\cdot s+e^{{}^{\prime }}+P\cdot s^{2}\;mod\;P\cdot Q$$

The above auxiliary computation key will be used in the multiplication, so at least the computation side should hold the key. The encryption function Encrypt is also run by the cloud server, as follows.

Generate $r\leftarrow \chi_{r}$ and $e_{0},\;e_{1}\leftarrow \chi_{e}$ and encrypt the user data with Key to obtain the ciphertext CT (Ciphertext). *m* is a plain text polynomial, and m∈R.

(9) $$c\leftarrow r\cdot pk+\left(m+e_{0},\;e_{1}\right)\;mod\;Q$$

(3) The similarity formula for wireless signal strength is pre-stored in the cloud server, which is represented here by the function *f*. In addition, the cloud space also runs an evaluation function Evaluate. The data processing method *f* given by the user operates on the cipher text so that the result is equivalent to the encryption of $f\left(Data\right)$ by the user with the key.
(4) The cloud space uses the function *f* to process wireless signals. The final result of the process is returned to the smartphone.
(5) The smartphone uses the decryption function Decrypt to process the processing result $f\left(Data\right)$ returned from the cloud space. The final result is the correlation coefficient value of the people’s trajectory data.

For the decryption operation, the cipher text $c\in R_{q}^{2}$ and the private key sk are needed to calculate the result and return it.

(10) $$\langle c,\;sk\rangle \;mod\;q=c_{0}+c_{1}\cdot s\;mod\;q$$

There are several encryption schemes in homomorphic encryption. In the subsequent similarity calculation, additive and multiplicative operations are involved. Therefore, it is important to choose the HE scheme that uses both additive and multiplicative homomorphism in this scheme.

Therefore, the similarity measure values are calculated using the public key pk and the cipher text $c_{1},\;c_{2}$.

In the calculation of homomorphic cipher addition, two ciphertexts $c,\;c^{{}^{\prime }}\in R_{q}^{2}$ are used to obtain the following result:

(11) $$c_{add}\leftarrow c+c^{{}^{\prime }}\;mod\;q,\;c_{add}\in R_{q}^{2}$$

In homomorphic cipher multiplication, multiplication can be performed between the ciphertext and plaintext and also between two ciphertexts. In this study, two ciphertexts are multiplied. Given two ciphertexts $c,\;c^{{}^{\prime }}\in R_{q}^{2}$, calculate and output the result:

(12) $$\left(d_{0},\;d_{1},\;d_{2}\right)=\left(c_{0}c_{0}^{{}^{\prime }},\;c_{0}c_{1}^{{}^{\prime }}+c_{1}c_{0}^{{}^{\prime }},\;c_{1}c_{1}^{{}^{\prime }}\right)\;mod\;q$$

(13) $$c_{mult}\leftarrow \left(d_{0},\;d_{1}\right)+\left\lfloor P^{-1}\cdot d_{2}\cdot evk\right\rceil \in \langle P^{-1}\cdot d_{2}\cdot evk,\;sk\rangle \;mod\;q,\;c_{mult}\in R_{q}^{2}$$

**Figure 3:**
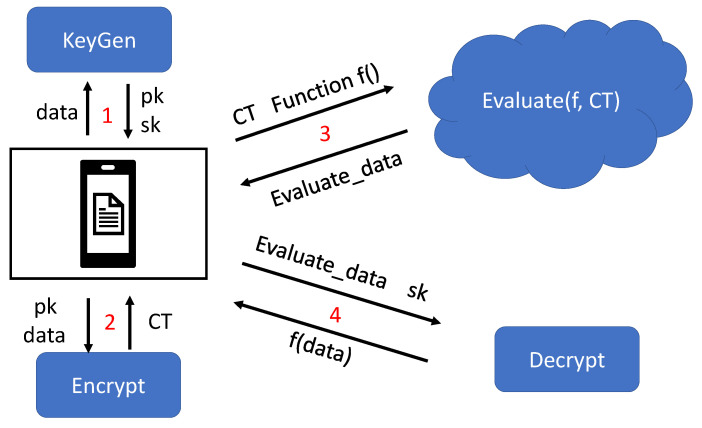
RSS Encryption Chart. Numbers 1-4 of the figure shows the steps for homomorphic encryption to process data. The first step generates public keys (pk) and private keys (sk). The second step uses the public key to encrypt the data. The third step uses ciphertext data (CT) and formulas (Function f()) to calculate and return ciphertext results (Evaluate_data). The fourth step uses the private key to decrypt the ciphertext result, and obtains the calculated result after decryption (f(data)).

The whole algorithm is designed with the idea of obfuscating the plaintext by the number of polynomials, modulo operations, and interactive operations. In the whole scheme, RSS is considered to be encrypted using a homomorphic encryption method to secure the data. Figure 3 shows the detailed steps of homomorphic encryption. Both the user’s dataset and similar results of trajectory matching degree are encrypted using the homomorphic encryption scheme. Thus, the confidentiality of the RSS dataset and the final result obtained can be attributed to the security of the homomorphic encryption algorithm. As shown in [48], the security of homomorphic encryption is determined by its own algorithm. The relevant result data obtained from the cloud are also not recoverable from the original data. The CKKS algorithm is a semi-contractual encryption scheme. For any given ciphertext, only the holder with the encryption key can decrypt that ciphertext. Thus, the security of homomorphic encryption is determined by the encryption key, which is in the hands of the holder and is thus more secure.

### 3.3. Similarity Calculation and Missing Value Complementation

#### 3.3.1. MAC Address Matching

After the data encryption operation, the privacy of the collected data is protected to a certain extent. Therefore, there will be no danger of privacy leakage for the next operation.

The same MAC address is hashed and encrypted, and its ciphertext remains the same. In this case, the first step is to match the wireless devices they detect. From this, we know whether A and B are in the same spatial location and thus match the MAC addresses in their messages.

To ensure consistency between time and space in the trajectory data, the same time period is partitioned first. After that, the MAC address data lists of two people are matched to find the same MAC addresses and arrange them in the same order. Before performing MAC address matching, the similarity of the text of the MAC address list should be calculated. If the similarity of the MAC addresses in the two messages is extremely low, there is no need to compare them one by one. It can be concluded from this information that the trajectory data of two people are not similar and exclude the possibility of contact. If this text information is too large, then the whole process becomes computationally intensive and time-consuming. However, this study was conducted for the data collected by a smart device. In fact, the number of wireless devices received around a fixed location is limited, so the study is feasible.

#### 3.3.2. Similarity Calculation

After the data are encrypted, the final step of similarity calculation can be performed. Processing the data by time and MAC address attributes, a matrix block consisting of RSS will be formed [49,50]. The matrix block is composed of RSS obtained at the same time period and under the same router devices. In this study, the original ciphertext matrix block $X=\left[X_{1},X_{2}...\right.\left.X_{n}\right]$ will be split into multiple small matrix blocks:

$$X_{1}=\left[\begin{array}{c} x_{11}x_{12}\cdots x_{1n}\\ x_{21}x_{22}\cdots x_{2n}\\ \cdots \\ x_{n1}x_{n2}\cdots x_{\mathrm{nn}} \end{array}\right]X_{2}=\left[\begin{array}{c} x_{11}x_{12}\cdots x_{1n}\\ x_{21}x_{22}\cdots x_{2n}\\ \cdots \\ x_{n1}x_{n2}\cdots x_{\mathrm{nn}} \end{array}\right]\cdots X_{n}=\left[\begin{array}{c} x_{11}x_{12}\cdots x_{1n}\\ x_{21}x_{22}\cdots x_{2n}\\ \cdots \\ x_{n1}x_{n2}\cdots x_{\mathrm{nn}} \end{array}\right]$$

**Figure 4:**
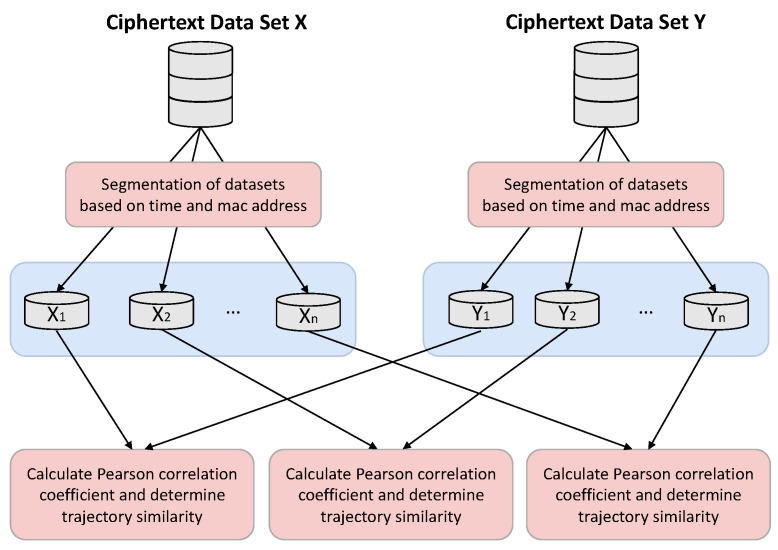
Segmentation Calculation Process for Determining the Similarity of Trajectories.

In addition, the above matrix is based on a matrix with the same ranks obtained from two conditional attributes, time and MAC address. As is shown in Figure 4, this split matrix block will facilitate the calculation of Pearson’s correlation coefficients. This is a method that can better determine similarity. It tends to give better results when the data are not very standardized. Ultimately, the result of this calculation can be used as a measure of trajectory data similarity.

The Pearson correlation coefficient between two variables is defined as the quotient of the covariance and standard deviation between the two variables. The Equations (14) and (15) are used to calculate the Pearson correlation coefficient. *X* and *Y* represent the two sets of data involved in the calculation. *N* represents the length of the data:

Formula 1:

(14) $$\rho_{X,\;Y}=\frac{N\sum XY-\sum X\sum Y}{\sqrt{N\sum X^{2}-{\left(\sum X\right)}^{2}}\sqrt{N\sum Y^{2}-{\left(\sum Y\right)}^{2}}}$$

Formula 2:

(15) $$\rho_{X,\;Y}=\frac{cov\left(X,Y\right)}{\sigma_{X}\sigma_{Y}}=\frac{E\left(\left(X-\mu_{X}\right)\left(Y-\mu_{Y}\right)\right)}{\sigma_{X}\sigma_{Y}}=\frac{E\left(XY\right)-E\left(X\right)E\left(Y\right)}{\sqrt{E\left(X^{2}\right)-E^{2}\left(X\right)}\sqrt{E\left(Y^{2}\right)-E^{2}\left(Y\right)}}$$

The current homomorphic encryption algorithm can perform additive and multiplicative homomorphic operations. In addition, the Pearson similarity coefficient formula involves the covariance and standard deviation of the sample data. So, the ciphertext after homomorphic encryption cannot be calculated directly by the Pearson similarity algorithm. Considering the limitations of homomorphic encryption, this section proposes an algorithm for computing the Pearson correlation coefficient [51,52]. See Algorithm 1 for detailed steps. The mean value between the matrices is first calculated by the homomorphic addition operation and homomorphic multiplication operation, and then the value is calculated by the dot product. After that, the Pearson correlation coefficient formula is used to obtain the similarity values.   
**Algorithm 1:** Algorithm for calculating Pearson’s correlation coefficient for ciphertext
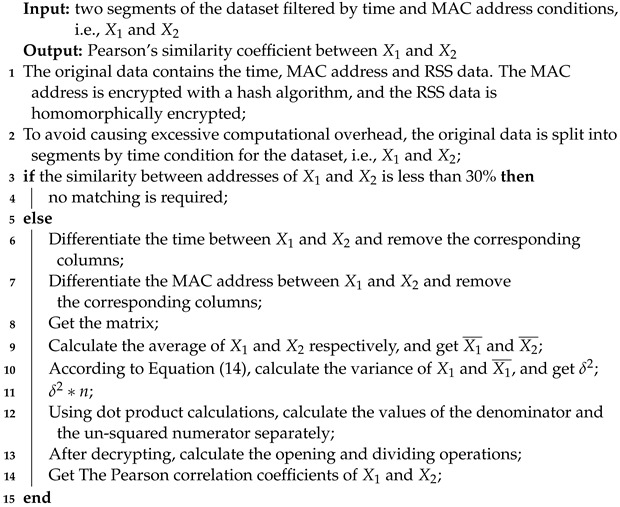


#### 3.3.3. Missing Value Completion Based on Homomorphic Encryption

**Figure 5:**
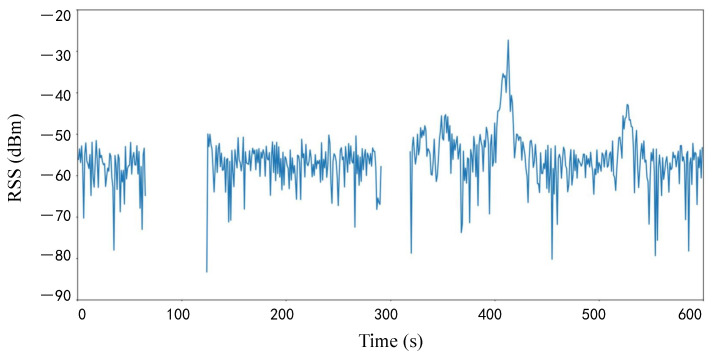
RSS Change Chart.

During the data collection, another problem was encountered that would affect the final results. Figure 5 presents a line graph of the RSS received from a particular router over time during the experiment. It is clear from Figure 5 that the wireless signal strength is null at some points in time. This indicates that RSS was not acquired at these time points. There are many reasons for missing RSS data, such as instability of the wireless router itself, Wi-Fi signal congestion, differences in connected devices, and obstacles in the transmission process. The calculating similarity in the case of missing data can easily degrade the accuracy and affect the final experimental results.

In order to ensure the accuracy of the experimental results, the missing data need to be processed for completeness. Mean, linear regression, and KNN methods were used here to complement the missing values. These methods were used in the experimental phase.

(1) Mean Padding Based on Homomorphic Encryption

In the context of statistical methods, numerical data are usually complemented using methods such as mean values. The original data are encrypted by the CKKS scheme, which changes the original data type. Therefore, it is necessary to study the calculation of the mean value in the homomorphic encryption state and in the data completing problem.

The basic idea of the homomorphic encryption-based mean missing value completion algorithm is as follows. Each value in the dataset is encrypted using homomorphic encryption, and missing values are replaced with zeros or other default values. Then, the homomorphic cryptographic addition operation is performed on the encrypted dataset. First, the sum of the data in each column of the ciphertext form is obtained, and then the sum of the ciphertext and the length of the ciphertext are calculated to obtain the average value of the ciphertext form. Finally, the missing values are replaced by the average value to obtain the complemented complete dataset.

(2) Linear Regression Complementation Based on Homomorphic Encryption

It has been mathematically shown that any dataset can be computed using a linear regression equation [53]. Since there is no actual correspondence between RSS and time, a segmented regression is used to perform additional operations on the dataset.

In order to make the additive isomorphism calculation applicable to linear regression analysis, the corresponding equivalent transformation of the equations for calculating the regression parameters is required.

An important task in analyzing linear regression is to determine the regression equation Y=α+βX. The least-squares method is commonly used to determine the parameters α and β, which are calculated as follows:

(16) $$\alpha \;=\;\frac{1}{n}\sum_{i=1}y_{i}-\beta \frac{1}{n}\sum_{i=1}x_{i}$$

(17) $$\beta \;=\;\frac{n\sum_{i=1}^{n}x_{i}y_{i}-\sum_{i=1}^{n}x_{i}\sum_{i=1}^{n}y_{i}}{n\sum_{i=1}^{n}{x_{i}}^{2}-{\left(\sum_{i=1}^{n}x_{i}\right)}^{2}}$$

The encrypted dataset is recorded as $X=\left[X_{1},X_{2}...\right.\left.X_{n}\right]$. The encrypted dataset *X* is uploaded to the cloud server, and then the server runs a linear regression equation on the encrypted dataset and finally obtains the prediction result. This result is used to complete the processing of missing values. During the whole training process, the server has no access to the information of the dataset, which ensures that the user’s privacy is not violated.

(3) KNN Complementation Based on Homomorphic Encryption

Machine learning [54] was also used to complement the dataset. Considering the practical situation, the space size is limited to a mall or large building so that the activity range and routes are basically countable. In this way, the training set can be continuously expanded and improved, and the missing data caused by some objective factors can be made up. In the case that the training set happens to be perfect, the missing data are calculated as Euclidean distances from the data in the training dataset. When *k* is taken as 1, the training set is the output of trajectory data in the training dataset. These trajectory data complete the missing values in the original dataset. Objectively, it is more realistic to use KNN to complement the missing values. The use of homomorphic encryption ensures that the user’s private data are not accessed. After that, with the help of the cloud-based KNN computational model, the trajectory matching results expected by users can be obtained. In practical application scenarios, some improvements to the KNN algorithm are needed to better adapt to the trajectory matching scenario. The traditional nearest-neighbor formula starts by calculating the Euclidean distance from the test point to each point in the dataset. The formula is expressed as follows, where *l* is the original number of each value in the trajectory data:

(18) $$d\left(x_{i},\;x_{j}\right)=\sqrt{{\sum_{l=1}^{n}\left(x_{l}^{i}-x_{l}^{j}\right)}^{2}}$$

It is difficult to perform the open-square operation under homomorphic encryption conditions. In this study, we propose the use os the square function instead of the quadratic square operation to calculate the Euclidean distance between data in the verification set and the training set. This ignores the open-square operation under ciphertext conditions. It not only improves the overall operation speed of the algorithm, but also improves the accuracy of the algorithm, making it more suitable for cloud computing and other fields.

## 4. Experiments and Results

The purpose of this experiment is to verify the effectiveness of the aforementioned encryption method. The study was conducted by collecting users’ trajectory data in different basic scenarios and performing similarity calculations on the ciphertext. In addition, for the missing values in the collected data, the study adopts the mean, linear regression equation, and KNN to complement the dataset. The accuracy of the completed data will be more favorable for representing users’ real trajectory data.

### 4.1. Design of Experiments

To verify the effectiveness of the scheme and its advantages, RSS data acquisition, data processing, and privacy calculation related to homomorphic encryption are studied.

To verify the precision loss of encrypted data calculations, relevant experiments are set up. Comparative experiments are conducted using 36 sets of trajectory data, which are divided into encrypted and unencrypted states. The precision loss value of homomorphic encryption on the data is obtained by comparing the matching results.

An experiment is set up to calculate the similarity of crowd trajectories. The Pearson correlation coefficient is used to calculate the similarity coefficient of the trajectory data.

In the experiment on complementing missing values, some data are selected for the experiment. There are three ways to complement the missing values in this study: mean, linear regression equation, and KNN. Each method calculates the similarity of its complementary data to the original data. Ultimately, we know which is the most accurate way to complement missing data.

### 4.2. Experiment Preparation

The feasibility of this research proposal is verified by providing feasible experiments. Two laptops are used as servers in this experiment. One is used as a dedicated server for data collection, specifically for receiving data and performing encryption and decryption operations. The other is a cloud server for encrypted data processing and similarity calculation operations. In addition, several cell phones were used as clients for RSS collection. All laptops ran the Windows 10 operating system and needed to download and install Android Studio. All smartphones needed to download and install the data collection applet. The applet collects nearby RSS and sends the collected data as a time-named file to the server for data collection. The dataset records the trajectory data of people. The applet runs on the smart device, takes up little memory, is fast, and can collect data automatically every 1 s or manually on its own at any time. The mobile application written using Android Studio was designed to take advantage of the portability of smartphones and to more easily collect RSS data around them. For data privacy protection, the TenSEAL library in python is used to make calls on when to implement the homomorphic encryption algorithm.

**Figure 6:**
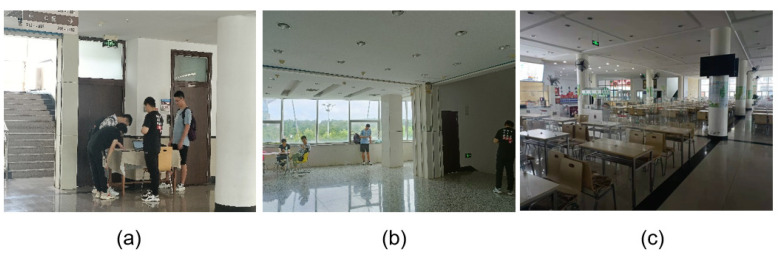
Different experimental scenarios are shown in the figures. (**a**) shows the experimental configuration and data acquisition in the teaching area. (**b**) is data acquisition in the complex building. (**c**) is data acquisition in the school canteen.

To demonstrate the feasibility of experimental scenarios, the entire experimental platform was built and experiments were conducted in different scenarios. As shown in Figure 6, Figure 6a is a teaching building frequented by teachers and students. Figure 6b is a large open work area with many wireless devices. Figure 6c is a school canteen with a relatively large population. In the experiment, there are four variables: scene, dispersion of people, sampling rate, and sample size. Experimental data were collected using each of the possible combinations of these four variables. Similarity calculations are then performed using the similarity formula to obtain experimental results. The results will be analyzed in Section 4.4.

### 4.3. Experimental Procedure

The experiments are conducted in different scenarios in which people’s trajectories are randomly varied. In each scenario, there are five people walking around, each carrying a smartphone. The smartphone is equipped with the applet, which, when opened, automatically collects the RSS around it and sends it off. The time interval for which the packets are automatically sent off is set to 1 s. In addition, the sample rate and sample size are set to 60 packets/min and 5 min, respectively. The sample rate indicates the number of packets sent from the smartphone to the server per minute, and the sample size indicates the time for collecting the data.

The data collection process is simple and easy, using mainly devices such as routers and smartphones. Data can be collected in crowded places such as restaurants and shopping malls, and the collection places are diverse. In the actual experiment, people choose their walking directions in different scenarios such as the school canteen, the teaching building, and the work area. They sometimes walk together and sometimes walk far apart from each other. Based on the collected information, people’s trajectory data can be roughly derived. After a series of operations such as encryption, calculation, and decryption by the server, the final experimental results are obtained.

In addition, after obtaining the real trajectory data, the data are processed. Inevitably, some data are missing during the processing of the data. In the processing of the data, the missing data are simply ignored and not calculated. If the amount of data is quite large and the amount of missing data is small, the missing data do not have much impact on the final result. However, when the amount of missing data is too large, it will have an impact on the results. Therefore, the missing values need to be complemented. In the process of calculating the missing values, three methods are successively used. The first is the averaging method, the second is the linear regression method, and the third is the machine learning method, which mainly uses KNN for calculating missing values.

### 4.4. Experimental Results

#### 4.4.1. Handling of Missing Datasets

(1) Average Method

For numerical data, the average of the missing values is counted by the number of non-missing values and the sum of non-missing values.

A segment of RSS data is taken for mean completion. According to the calculation, the sum of the data is −1834.426 and the length of the data without missing values is 21, so the average value obtained is −87.354. The similarity of this trajectory data with the original trajectory data is 98.1%. So, when the interval of the missing data segment does not exceed (0, 1), the accuracy of the method of filling the missing values with the average is very high. However, there is a potential problem with using the average to complement the missing values. If the missing data interval of the original dataset is large, the final result is not good. For example, at (10, 50), the mean value calculated is 30. After complementing the data using the average, the similarity of this dataset to the original dataset is 50%. Therefore, the method of using the average value to complement the missing data values is not valid when there is a large segment of missing data in the dataset.

(2) Linear Regression Equation

The linear regression equation is obtained by jointly calculating the original data and the data in $x=\left[1,2,3,4,\ldots \right]$. The linear regression function for this dataset is y=0.13x−89.15. The y-value corresponding to the current missing value can be calculated. Thus, the missing value to be filled is $\left[-87.60,-87.47,-87.34,-87.20,-87.07,-86.94\right]$.

There are some advantages to using linear regression to complement missing values compared to mean calculation. Linear regression takes into account the correlation between individual features and can predict missing values more accurately. On the contrary, the mean value calculation does not consider the influence of other features on the missing values, so the prediction results may not be accurate enough.

(3) Machine Learning

**Figure 7:**
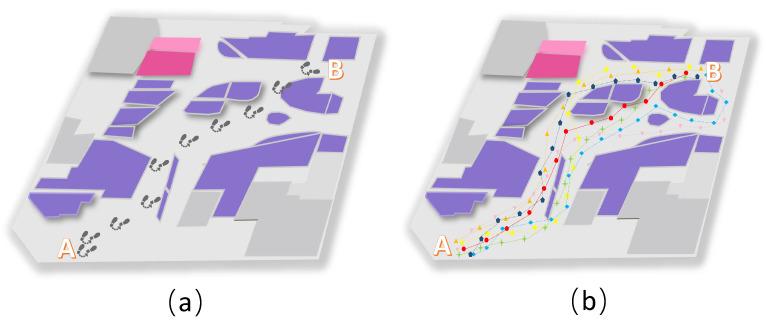
The graph is a complementary graph of the dataset using machine learning. (**a**) represents the actual trajectory of a person. (**b**) denotes the (partial) dataset after machine learning, and each colored line in the figure represents a group of trajectory data.

**Figure 8:**
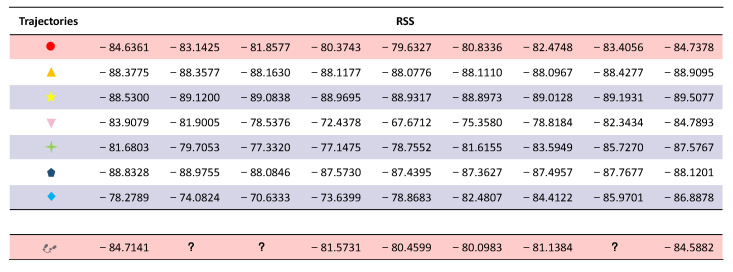
Path Matching Results. The mark “?” represents a missing RSS value.

There will be multiple paths from point A to point B. Figure 7b lists some trajectory paths. Figure 7a shows the path that someone usually chooses to go from point A to point B. The signal strength data of a person from point A to point B are missing. These data can be calculated using machine learning in the corresponding dataset. After that, the segment of the dataset that best matches the current missing dataset will be output, and part of the calculation results are shown in Figure 8. According to the Pearson calculation, the similarity between the red trajectory and the actual trajectory is 99.3%. The similarity between the orange trajectory and the actual trajectory is 87.8%. The similarity between the yellow trajectory and the actual trajectory is 84.1%. The similarity between the pink trajectory and the actual trajectory is 85.3%. The similarity between the green trajectory and the actual trajectory is 95.8%. The similarity between the brown-green trajectory and the actual trajectory is 85.8%. The similarity between the blue trajectory and the actual trajectory is 76.9%.

Therefore, the red dot path in Figure 7b has the highest matching value with the path data in Figure 7a. So, this dataset is closest to the current trajectory of the user. The signal data with red dots in Figure 7b can be output to complement the current missing dataset. Figure 8 shows an image of the data after complementing the missing values using the KNN obtained above. The similarity of this trajectory data with the original trajectory data is 99.1%.

Linear regression only considers the linear relationship between features. In contrast, the KNN algorithm can predict missing values by finding the records that are most similar to the actual trajectory data and using the values of these records. Therefore, the KNN algorithm does not depend on the distribution of the data. For the case of missing values, the KNN algorithm has little effect on the overall prediction results. Therefore, KNN has better robustness.

#### 4.4.2. Similarity Calculation

The trajectory data of users obtained without encryption can clearly reflect their location. After performing a series of encryption operations on the trajectory data, the final information obtained is essentially the same as that obtained without encryption. Thus, the feasibility of this encryption scheme is demonstrated.

As shown in Table 3, the results of the similarity experiments based on encrypted and unencrypted states are calculated.

From the table, we can see that the similarity of a group of trajectory data measured by users at a distance of 10 m has a precision difference of 0.0000276 between encrypted and unencrypted states. For the similarity of a set of trajectory data measured at a distance of 1 m, the difference between the two states is 0.000001545. There is no practical effect on the determination of the results, so it is negligible.

**Figure 9:**
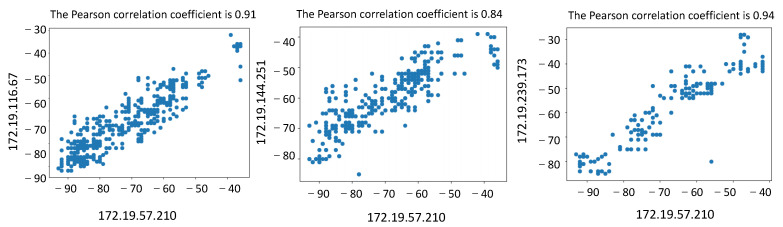
Calculation of Pearson’s correlation coefficient at a user distance of 1 m.

**Figure 10:**
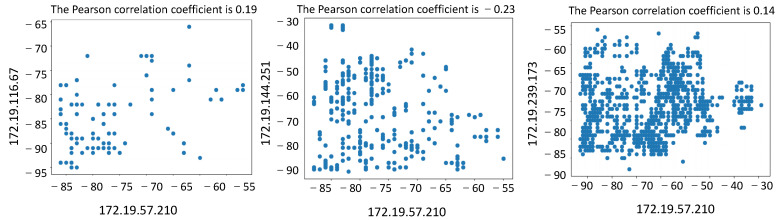
Calculation of Pearson’s correlation coefficient at a user distance of 10 m.

In the trajectory matching experiment, the trajectory data in the school building, complex building, and cafeteria scenes were selected for matching calculation. The distances between users were set at 1 m and 10 m, respectively. The analysis of the experimental data shows that when the distance is about 1 m, the value of the Pearson correlation coefficient obtained is above 0.80, as shown in Figure 9. The Pearson correlation coefficient is as low as 0.20 when the distance is set to 10 m, as shown in Figure 10. Therefore, it is feasible to use RSS signals for trajectory matching based on the calculation of actual trajectory data.

#### 4.4.3. Complexity Analysis

Multiple algorithmic processes are involved in the study. The SHA-256 algorithm is used to encrypt the MAC address for anonymization, and the whole process involves several iterations of the loop, so its time complexity is O (*n*). Homomorphic encryption is divided into the homomorphic addition operation and the homomorphic multiplication operation. The time complexity of the homomorphic addition operation is O (*n*), while the time complexity of the homomorphic multiplication operation is O ($n^{2}$). However, after utilizing the number round transformation, the time complexity of the multiplication operation is O ($n\log_{2}n$). The time complexity is O ($n^{2}$) when calculating the Pearson correlation coefficient between different data. In the ciphertext state of completing the missing values, the time complexity of calculating each segment of missing data using both mean and linear regression methods is O (1). Finally, the time complexity of using KNN to complete the missing data is O (*n*).

In addition, the space complexity of the homomorphic encryption algorithm in the ciphertext state is generally 4–5 orders of magnitude higher than that in the plaintext state, but the segmentation calculation in this study reduces the storage pressure of the server. In the process of completing the missing values, a new space is needed to store the computed dataset, so the space complexity is O (*n*).

## 5. Discussion

Designing an effective and secure trajectory matching algorithm is a major challenge. Thus, this research implements an anonymous trajectory matching computation method and expands the application scenarios of homomorphic encryption algorithms. Based on the hash algorithm and homomorphic encryption algorithm, the trajectory matching scheme supporting addition and multiplication operations on RSS is realized. This study not only ensures the privacy and security of users’ original trajectory data, but also completes the trajectory matching computation. In addition, the final similarity results are analyzed from the perspectives of encryption time and accuracy loss. The proposed algorithm can also perform complex similarity calculations in ciphertext, obtaining good results.

In addition, while processing the data, it was found that there were some missing values in the collected dataset due to problems such as interference from indoor obstacles. Thus, the mean value algorithm, linear regression algorithm, and KNN algorithm, which can complement the missing values in the ciphertext, were employed. In addition, the original data and the complemented dataset are provided for evaluation. The accuracy of the complemented dataset and the trajectory matching results are compared.

However, the research has certain flaws and shortcomings. In the future, research on the methods of this topic will be further optimized.

First, the homomorphic encryption algorithm used in the trajectory computation scheme has a sufficiently large memory overhead. The current memory overhead required to compute the similarity of trajectory segments is sufficient for an ordinary CPU to run. However, larger volumes of data will cause the problems of a high memory overhead and inefficient computation. Therefore, improving the efficiency of ciphertext computation and solving memory overhead issues are problems worth investigating. Second, the homomorphic encryption technique is used in the trajectory computation scheme to achieve privacy protection of the original data in this paper. More privacy-preserving techniques, such as differential privacy and homomorphic signatures, could be explored in the future. The effectiveness and feasibility of privacy-preserving methods can be improved. Third, the trajectory computing scheme proposed in this study is a special computing task, and the use of this scheme for privacy protection can be applied to a wider range of computing scenarios in the future, such as machine learning, data mining, and other fields.

## 6. Conclusions

In the trajectory matching process, the similarity coefficients between a large amount of data need to be calculated on a cloud platform with a large amount of storage resources and sufficient computing power. In addition, there may be some missing values in the dataset due to the existence of problems such as interference from indoors obstacles. To solve the above problems and challenges, this paper proposes a homomorphic encryption-based trajectory computation scheme and provides three methods to complement missing values in the ciphertext. Overall, the experimental results in this paper fully verify that the method has some advantages in practical applications. In the future, the scheme can be further improved and optimized through verification and application in more datasets and practical scenarios. We have also explored more optimization technologies for privacy protection and encrypted computing to improve data privacy protection and computing efficiency.

## Figures and Tables

**Table 1:** Advantages and disadvantages of indoor positioning signal source.

Signal Source	Advantages	Disadvantages	Related Work
RFID	High positioning accuracy, contactless automatic identification, suitable for indoor positioning tracking of items, such as production, storage logistics, etc.	High cost, limited to identifying items in close proximity, and susceptible to interference; not used for positioning in large areas.	Ma Y, Hui X, Kan E C. 3D real-time indoor localization via broadband nonlinear backscatter in passive devices with centimeter precision [26].
Light Sensing Technology	High reliability, no RF interference.	Must have a sufficient light source and is more sensitive to changes in the environment.	Zhang C, Zhang X. LiTell: Robust indoor localization using unmodified light fixtures [27].
Bluetooth	High positioning accuracy, fast positioning speed, low power consumption.	Bluetooth devices need to be deployed and Bluetooth signals may be tapped or attacked.	Okşar İ. A Bluetooth signal strength-based indoor localization method [28].
Wi-Fi	Fast speed, wide radio wave coverage, and no need to deploy dedicated gateway equipment.	Adequate Wi-Fi signal base station coverage is required, and the signal is susceptible to interference indoors.	This signal source is used in this study.

**Table 2:** Advantages and disadvantages of location privacy-related algorithms.

Algorithm	Advantages	Disadvantages	Related Work
K-anonymity algorithm	Concern for data privacy and integrity, simple to implement.	Unable to handle correlated data and large data loss.	Samarati P, Sweeney L. Generalizing data to provide anonymity when disclosing information. [22]
Differential privacy algorithm	Effectively prevents targeted attacks and makes it difficult to infer users’ private information from others’ information.	Randomly adding noise not only increases the computational requirements, but also affects the accuracy of the data.	Yin C, Xi J, Sun R, et al. Location privacy protection based on differential privacy strategy for big data in industrial internet of things [23], etc., [29,30,31]
Homomorphic encryption [32,33,34,35,36]	Participate in the calculation in the encrypted state.	For large-scale datasets, a large amount of computational resources are consumed.	This algorithm is used in this study.

**Table 3:** Similarity calculation results (Partial results).

Distance among People (m)	Pearson Correlation Coefficient for Unencrypted Data	Pearson’s Correlation Coefficient for Encrypted Data
0	0.9067584202944	0.9067587979448
0.5	0.7347918294219	0.7347933742293
1	0.5415083176462	0.5415113253752
10	0.1373338630103	0.1373376528433

## Data Availability

The data presented in this study are available on request from the corresponding author.
